# Individual‐level leaf trait variation and correlation across biological and spatial scales

**DOI:** 10.1002/ece3.7425

**Published:** 2021-03-18

**Authors:** Feng Jiang, Marc W. Cadotte, Guangze Jin

**Affiliations:** ^1^ Center for Ecological Research Northeast Forestry University Harbin China; ^2^ Department of Biological Sciences University of Toronto Scarborough Toronto ON Canada; ^3^ Ecology and Evolutionary Biology University of Toronto Toronto ON Canada; ^4^ Key Laboratory of Sustainable Forest Ecosystem Management‐Ministry of Education Northeast Forestry University Harbin China

**Keywords:** intraspecific trait variation, leaf economics traits, leaf size traits, sample size, trait correlation, trait trade‐off

## Abstract

Even with increasing interest in the ecological importance of intraspecific trait variation (ITV) for better understanding ecological processes, few studies have quantified ITV in seedlings and assessed constraints imposed by trade‐offs and correlations among individual‐level leaf traits. Estimating the amount and role of ITV in seedlings is important to understand tree recruitment and long‐term forest dynamics. We measured ten different size, economics, and whole leaf traits (lamina and petiole) for more than 2,800 seedlings (height ≥ 10 cm and diameter at breast height < 1 cm) in 283 seedling plots and then quantified the amount of ITV and trait correlations across two biological (intraspecific and interspecific) and spatial (within and among plots) scales. Finally, we explored the effects of trait variance and sample size on the strength of trait correlations. We found about 40% (6%–63%) variation in leaf‐level traits was explained by ITV across all traits. Lamina and petiole traits were correlated across biological and spatial scales, whereas leaf size traits (e.g., lamina area) were weakly correlated with economics traits (e.g., specific lamina area); lamina mass ratio was strongly related to the petiole length. Trait correlations varied among species, plots, and different scales but there was no evidence that the strength of trait relationships was stronger at broader than finer biological and spatial scales. While larger trait variance increased the strength of correlations, the sample size was the most important factor that was negatively related to the strength of trait correlations. Our results showed that a large amount of trait variation was explained by ITV, which highlighted the importance of considering ITV when using trait‐based approaches in seedling ecology. In addition, sample size was an important factor that influenced the strength of trait correlations, which suggests that comparing trait correlations across studies should consider the differences in sample size.

## Graphical abstract

Using individual‐level leaf traits of seedlings, we found intraspecific trait variation was large and correlations were strong between lamina and petiole traits across and within species. Trait correlation strength varied across biological and spatial scales and with sample size.

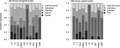

## 1 INTRODUCTION

Numerous and distinct plant species are distributed into forests around the world and show immensely diversified characteristics. Some of these characteristics are often envisioned by ecologists as functional traits due to their effects on plant growth, survival, and reproduction (Violle et al., 2007). Trait‐based approaches are preferred over taxonomic diversity measures because traits are believed to provide a predictive basis to understanding how ecological mechanisms influence community diversity and structure (Cadotte et al., 2013; Keddy, 1992; Laughlin, 2014; McGill et al., 2006). Consequently, there is an increasing interest in using species traits to better understand the nature of species ecological strategies and the constraints and trade‐offs that limit ecological opportunity (Diaz et al., 2016; Wright et al., 2004), variation in demographic rates (Pu et al., 2020; Visser et al., 2016; Wright et al., 2010), and overall community assembly (Cadotte et al., 2013, 2019). The species‐level traits used in the vast majority of trait‐based analyses explicitly or implicitly assume that interspecific trait variation is much larger than intraspecific trait variation (ITV). However, increasing numbers of studies suggest that the amount of ITV is comparable to that of interspecific trait variation (Albert et al., 2012; Messier et al., 2010; Siefert et al., 2015), and accounting for ITV can improve our understanding of community assembly (Chalmandrier et al., 2017; Jiang et al., 2018).

While most studies examining ITV have focused on plants at later life stages, for example, trees with a diameter at breast height (dbh) greater than 10 cm (Messier et al., 2010), mature coffee trees (Martin et al., 2017), and adult trees (Umaña & Swenson, 2019), there is little quantification of ITV and its importance at the seedling stage in natural systems. Seedlings represent an important bottleneck stage for survival because they are especially susceptible to mortality caused by weather events, natural enemies, and limited light availability (Augspurger, 1984; Comita et al., 2009). Seedling traits can influence their response to these pressures (Umaña et al., 2018), and trait‐mediated seedling dynamics will eventually influence the entire structure and composition of forest communities. Given the same spatial scale, the amount of seedling ITV is expected to be larger than adult trees due to their fast growth and the effects of spatial and temporal variation in understory microhabitats (Niinemets, 2010).

In understory environments with limited light availability, the leaf is an important plant organ for seedlings for intercepting light and photosynthesis. Assimilation products in leaves can be transported to other plant parts for functions such as growth or defense against herbivory. The most frequently evaluated leaf traits are the economics spectrum traits, which forms a trade‐off across species from a resource acquisitive strategy with short leaf lifespan and fast resource return to a resource conservative strategy with long leaf lifespan and slow resource return (Reich et al., 1997; Reich et al., 1999; Wright et al., 2004; Wright et al., 2005; but see Osnas et al., 2013). Many leaf traits are correlated with one another along this spectrum including leaf nitrogen content, leaf mass per area, and photosynthetic rate (Wright et al., 2004). However, recent broad‐scale studies find that leaf size is decoupled with economics traits (Diaz et al., 2016; Thomas et al., 2020). The correlations (or conversely, independence) between these leaf traits are important for plants and their ability to adapt to environmental gradients (Delhaye et al., 2020; Dwyer & Laughlin, 2017; Li et al., 2015). Studies that examine leaf economics usually measure leaf traits from the lamina only, but a whole leaf includes both lamina and petiole. While the lamina can intercept light, transport water and provide surface area for photosynthesis (Blonder et al., 2011; Lusk et al., 2019; Wright et al., 2004), the petiole can mediate the spatial position of a leaf and provide biomechanical support and hydraulic function (Poorter & Rozendaal, 2008). Evaluating the correlations between lamina and petiole traits can improve our understanding of trade‐offs among leaf traits at the whole leaf lens.

Given that the trait trade‐offs are usually examined using interspecific trait measurements across broad spatial scales, it is unknown if these trait correlations remain within species and at a local spatial scale (Anderegg et al., 2018; Fajardo & Piper, 2011; Liu et al., 2019; Martin et al., 2017; Messier et al., 2017; Niinemets, 2015; Umaña & Swenson, 2019; Wright et al., 2012). The strength of trait correlations is expected to be weaker within species and at a local spatial scale due to limited trait variance and trait‐specific responses to environmental gradients that filter trait variation nonrandomly (Messier et al., 2017). Some studies find weak correlations among lamina traits at the intraspecific level and at a local spatial scale (Anderegg et al., 2018; Wright et al., 2012). Conversely, other studies showed similar strength in trait correlations at the intraspecific level compared to species level or broad spatial scales (Hu et al., 2015; Martin et al., 2017; Niinemets, 2015). However, comparing these results is hampered because these studies include varied sample sizes and are conducted at different spatial scales, which likely influences trait variance and the strength of trait correlations (Anderegg et al., 2018; Wright et al., 2005). Therefore, studies are needed to evaluate whether trait correlations are weaker at finer biological and spatial scales, as well as whether sample size and trait variance influence these trait correlations.

**FIGURE 1:**
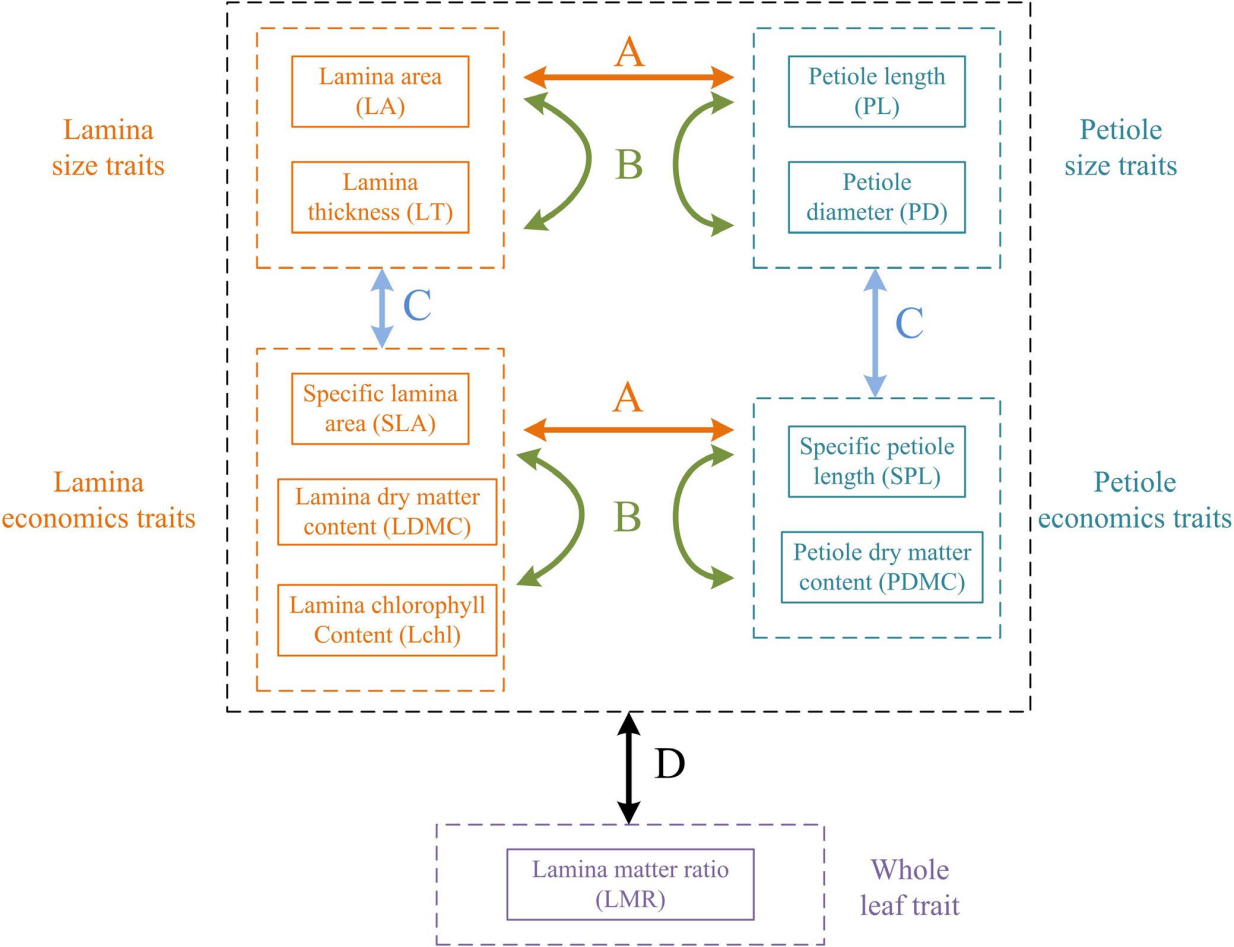
All leaf traits are divided into five groups: 1) lamina size traits, 2) lamina economics traits, 3) petiole size traits, 4) petiole economics traits, and 5) the whole leaf trait. These traits are hypothesized to show varying correlation strength among them: Traits can be correlated strongly (A) between the lamina and its corresponding petiole traits and (B) within each trait group; traits can be correlated weakly (C) across groups for lamina or petiole traits and (D) between the whole leaf trait and others

In this study, we measured 10 lamina and petiole traits for more than 2,800 seedlings of 30 broad‐leaved species in 283 seedling plots (4 m^2^). Such trait datasets, which include multiple species and a hierarchical sampling method, provide a good opportunity to explore whether trait correlations are weaker at finer scales and influenced by trait variance and sample size. We expect that a substantial amount of ITV exists at the seedling stage because of ontogenetic differences, plastic responses to micro‐environments and because trait variation has yet to be filtered out by abiotic and biotic influences. Specifically, we ask how biological (family, genus, species, individual, and leaf) and spatial (plot, species, individual, and leaf) scales explain the amount of trait variation. These two scales are believed to structure ITV in natural systems (Anderegg et al., 2018; Messier et al., ,,^2010^, ^2017^; Umaña & Swenson, 2019). While the biological scales refer to the relative importance of TIV among phylogenetic scales, the spatial scales examine the ITV along with environmental gradients (i.e., the plot level). We further explore whether leaf traits are correlated or decoupled between size and economics traits and between the laminar and petiole traits (the expectations of trait correlations are illustrated in Figure 1) and whether these correlations are influenced by biological or spatial scales. We expect that the strength of trait correlations is weaker at finer than broader scales. Finally, we assess whether the strength of correlations between leaf traits across different scales is influenced by trait variance and sample size. The strength of trait correlations is expected to be influenced by sample size and to increase with increasing trait variance.

## 2 MATERIALS AND METHODS

### 2.1 Study site

Our study was conducted in the Liangshui National Natural Reserve (47°10′50″N, 128°53′20″E) in Northeast China. The climate is temperate continental monsoon with most rainfall in summer. The mean annual temperature is −0.3°C, and the mean annual precipitation is 676 mm (Piao et al., 2013; Zhu et al., 2017). The mixed broad‐leaved Korean pine (*Pinus koraiensis*) forest is the most common vegetation type in Northeast China, and the Liangshui Reserve has a large area of these primary and undisturbed forests. In this Reserve, we established a 9 ha (300 m × 300 m) forest dynamics plot with no disturbance and all woody plants with the diameter at breast height (dbh) ≥ 1 cm were tagged, identified, mapped, and measured (dbh) (Condit, 1998).

### 2.2 Seedling plots and trait measurements

Within the 9 ha forest dynamics plot, we established a total of 900 4 m^2^ (2 m × 2 m) seedling plots (referred to as plots thereafter) at the intersections of a 10 m grid (Figure S1). In these seedling plots, we censused all woody plants with height ≥ 10 cm and dbh < 1 cm. This definition of seedling stage based on height and dbh is commonly used in global forest dynamics plots and broadly includes all plants at relatively early life stages (e.g., Comita et al., 2010). In August 2018, we collected leaf samples of all seedlings except for lianas and conifers in 283 plots distributed widely in the 9 ha forest dynamics plot (Figure S1). To minimize damage to seedlings, we only collected one leaf for small seedlings with few leaves, or two leaves for large seedlings at the end of the growing season. The healthy leaves with petiole were sampled and placed in foam boxes. Ice blocks were also placed in boxes to decrease the water loss of leaves. Then, these leaves were carried to the laboratory for trait measurements within 4 hr.

We measured a total of 10 leaf lamina and petiole traits and divided these traits into five groups (Figure 1): lamina size traits (lamina area, LA, cm^2^; lamina thickness, LT, mm*10), lamina economics traits (specific lamina area, SLA, cm^2^/g; lamina dry matter content, LDMC, g/g, lamina chlorophyll content, Lchl, mass‐based SPAD value), petiole size traits (petiole length, PL, cm; petiole diameter, PD, mm), petiole economics traits (specific petiole length, SPL, cm/g; petiole dry matter content, PDMC, g/g), and whole leaf economics trait (lamina matter ratio, LMR, g/g). LT was determined by a micrometer (0.01 mm) and then scanned to estimate LA. Fresh lamina and petiole were weighted by an analytical balance (0.0001 g) and then oven‐dried at 60°C to constant weight. SLA was the LA divided by lamina dry weight, and LDMC was the lamina dry weight divided by lamina fresh weight. Chlorophyll content per area was measured using the SPAD‐502 Plus meter (KONICA MINOLTA, INC) and multiplied by SLA to generate the Lchl (mass‐based measurement). PL was measured by a ruler (0.1 cm), and PD was determined by a micrometer (0.01 mm). SPL was calculated as PL divided by petiole dry weight, and PDMC was petiole dry weight divided by petiole fresh weight. Finally, LMR was the lamina dry weight divided by the whole leaf dry weight. There were three exceptions to the above methodology that affected how we sampled and processed leaves and trait measurements. First, some seedlings with no leaves or with only unhealthy leaves (e.g., yellowed) were not sampled. Second, if the leaves were partially grazed by herbivores (e.g., small holes), we used Photoshop CS6 to green the grazed parts to generate a more accurate estimate of LA. This corrected LA values were highly correlated with the original estimates (*R* ^2^ = .998). Third, for compound leaves, we used the leaflet for the trait measurements. But for LMR, we used all leaflets of a petiole. Before analyses, we excluded the observations with lamina and petiole dry matter < 0.0040 g (lamina: 16 observations; petiole: 3,207 observations) and petiole length ≤ 0.2 cm (262 observations). These very small observation values could bias some trait estimates because of the potentially large errors when we measured them using the analytical balance and ruler. Our final analyses included 5,185 leaves of 2,803 seedlings in 30 species in 283 seedling plots (Table S1).

### 2.3 Statistical analysis

We performed three analyses in this study, which corresponded to our three questions. First, we used a nested ANOVA with random effects to explore the variation of lamina and petiole traits explained by different biological (family, genus, species, individual, and leaf levels) and spatial scales (plot, species, individual, and leaf levels) (Messier et al., 2010). This analysis was performed using the *lme* and *varcomp* functions. Second, we used standardized major axis (SMA) regressions to evaluate the trait correlations illustrated in Figure 1. The SMA was used because we wanted to generate a scaling relationship between traits, and the traits as both responses and predictors had measurement errors. The *R* ^2^ from SMA regression was same as the square of *Pearson’ r*. The trait correlations were analyzed (a) between lamina and its corresponding petiole traits; (b) within trait groups; (c) across trait groups; and (d) between LMR and other traits (Figure 1). On the other hand, trait correlations were analyzed in four cases: (1) intraspecific level: We generated the average of each trait for each seedling and then calculated the correlations across individuals for each species; the common slope of SMA was tested across all species. (2) Species level: We generated the average of each trait for each species and then calculated the correlations across species. (3) Within plot level: We generated the average of each trait for each seedling and then calculated the correlations across individuals within each plot; the common slope of SMA was tested across all plots. (4) Plot level: We generated the average of all individuals in each plot for each trait and then calculated the correlations across plots. To understand whether the strength of trait correlations was weaker at finer scales, we used Student's *t* test to compare the slope and *R* ^2^ values of trait correlations within and across species (i.e., biological scales) and within and across plots (i.e., spatial scales). Sample size and trait variance were considered as weights separately in the *t* tests to account for their effects (Anderegg et al., 2018). The weighted *t* tests were performed using the *wtd.t.test* function in *weights* package. Species and plot with more than five seedlings were included in analyses (1) and (3). Third, we combined the results of correlations at species and plot levels (cases 1 and 3) to evaluate the effects of trait variance and sample size on the strength of correlation using multiple regression. The response variable was *R* ^2^ values of significant SMA regressions between traits, and the independent variables were variance of both traits, sample size, levels analyzed (species and plot levels), the interaction between trait variance and levels, as well as the interaction between sample size and levels. The trait variance and sample size were log‐transformed and standardized. All leaf traits were log‐transformed before our analyses. All analyses were performed in the R‐3.6.2 (R Core Team, 2020).

## 3 RESULTS

### 3.1 Variance partitioning

**FIGURE 2:**
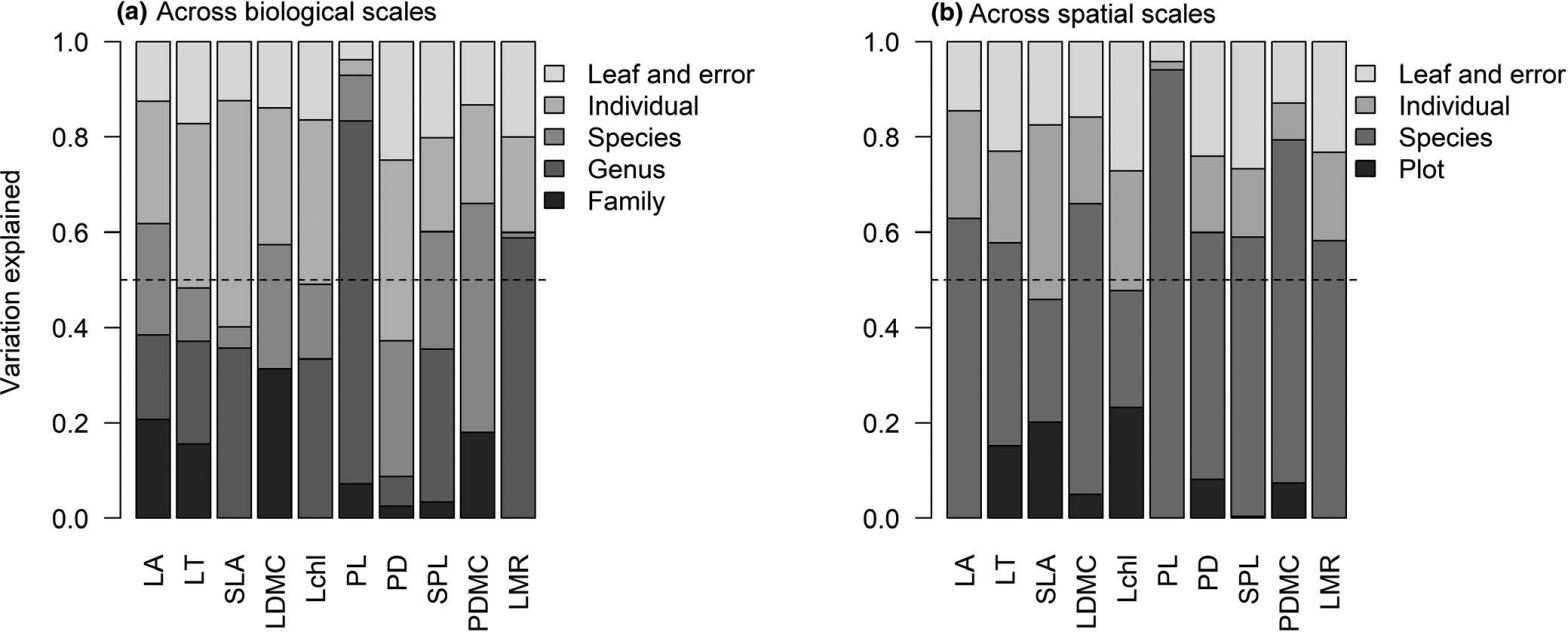
Results of variance partitioning for lamina and petiole traits across (a) biological and (b) spatial scales. The dashed line indicates the 50% of variation explained

Across the biological scales, variation explained by each level varied among leaf traits (Figure 2a). Specifically, variation explained by family varied across traits from 0% to 31.3%. Genus also accounted for a large amount of variation in PL (76.2%) and LMR (58.9%). The amount of variation explained by ITV varied from 7.1% (PL) to 62.8% (PD) with a mean of 42.7%. We found that >50% variation of two commonly used traits (SLA and LT) was explained by ITV. Across spatial scales, the relatively even variation of lamina traits was explained by different levels, whereas the most variation of petiole traits was mainly explained by species (Figure 2b). The amount of variation explained by ITV ranged from 6.0% (PL) to 54.1% (SLA) with a mean of 36.9%, which was similar as that across biological scales (Figure 2).

### 3.2 Correlations across biological and spatial scales

**FIGURE 3:**
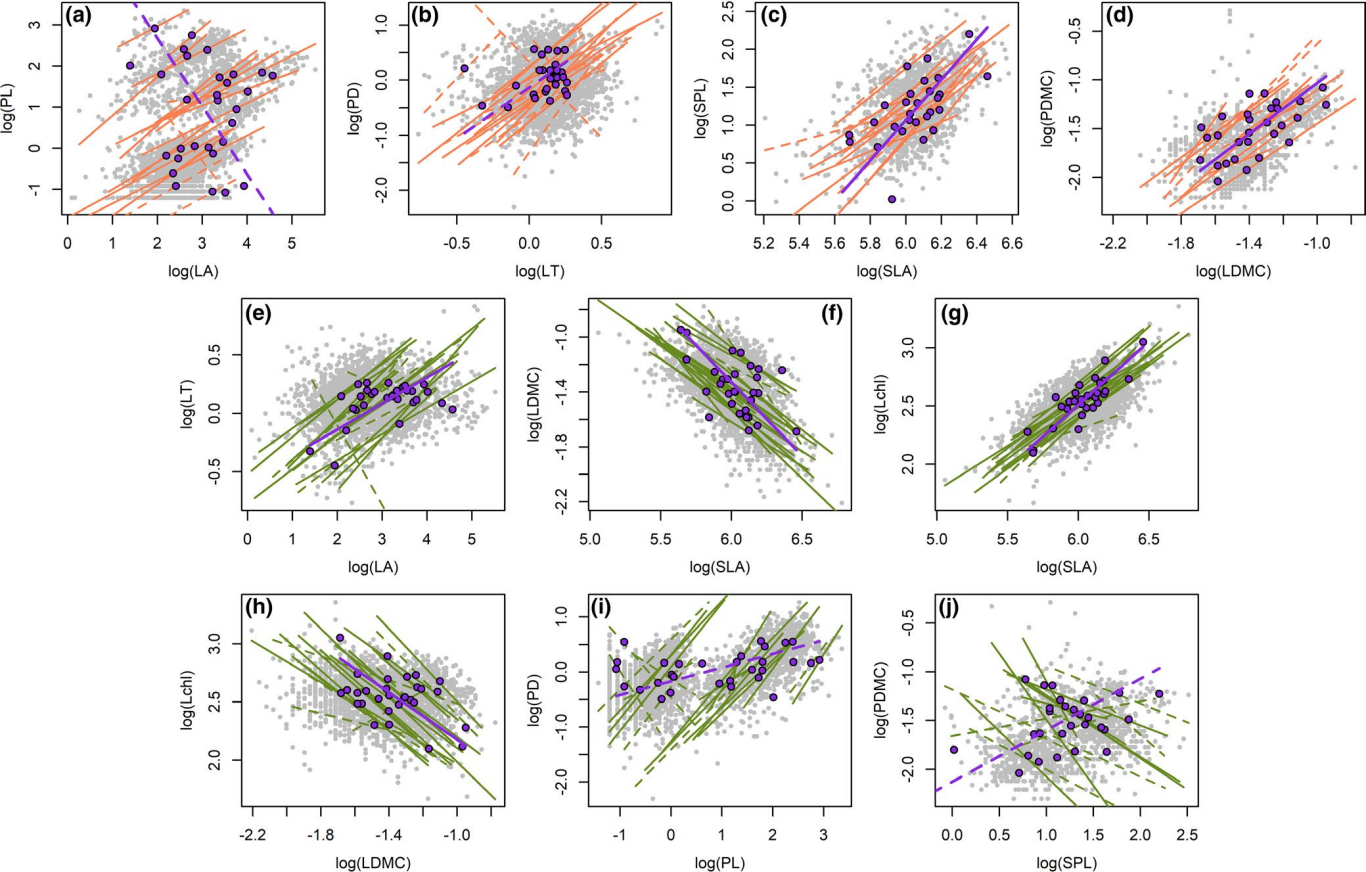
Correlations between lamina and petiole traits (a‐d, orange lines, Correlations A in Figure 1), and between leaf traits within trait groups (e‐j, green lines, Correlations B in Figure 1) within species and across species (purple lines). Grey circles, values of seedling individuals for all species; purple circles, mean values of species; dashed line, nonsignificant; solid line, significant. Correlations are generated using standardized major axis (SMA) regressions

Across biological levels, trait correlations varied within and across species (Figure 3, Figures S2,S3 and Table S2). First, between lamina traits and their corresponding petiole traits, correlations were positively correlated within most species (Figure 3a‐d), but only significant for economics traits across species (Figure 3c‐d). For PL versus LA, correlations were stronger within than across species (*t* test, Table S2). Second, within trait groups, correlations were strong for lamina traits at both levels (Figure 3e‐h). For LDMC versus SLA, the slope was steeper across than within species whereas the correlation was stronger within species; for Lchl versus SLA, the correlation was stronger across than within species (Table S2). Between petiole traits, correlations were significant for about half of species but nonsignificant across species (Figure 3i‐j). Third, across trait groups, correlations varied within and across species and were usually weak (Figure S2 and Table S2). While the correlations of SPL versus PD and PDMC versus PD were stronger across than within species, the slope of SPL versus PD was steeper across species (Table S2). For SPL versus PL, traits were positively correlated across species but negatively correlated within most species (Figure S2). Fourth, PL was the most correlated trait with LMR for both levels (*R* ^2^ = .68 across species, Figure S3). LMR showed stronger correlations across than within species against LT, Lchl, PL, and SPL (Table S2). Finally, there are no common slopes within species for all trait combinations (Table S2).

**FIGURE 4:**
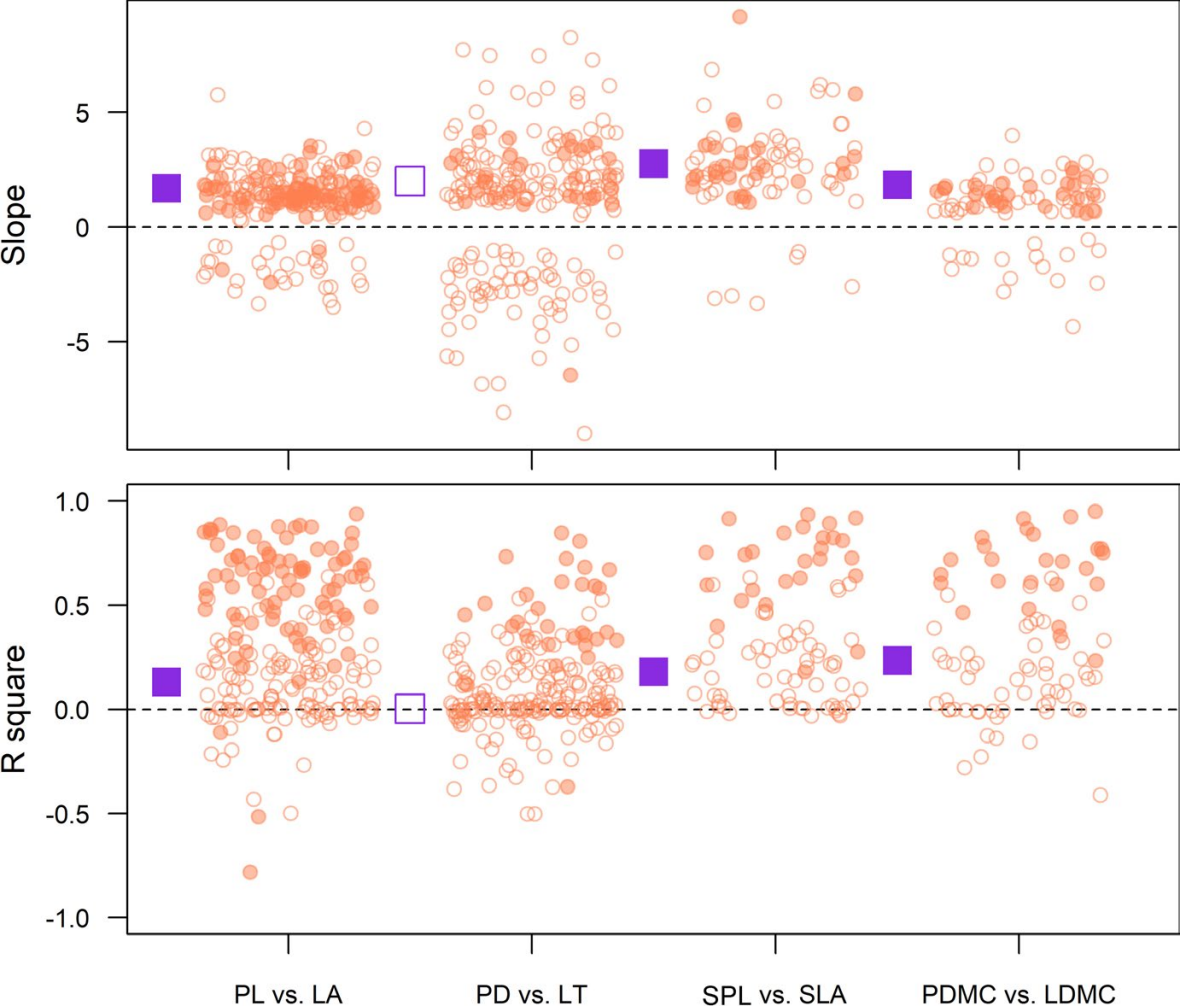
Slopes and *R* ^2^ values of standardized major axis (SMA) regressions between lamina and petiole traits within plots (circle) and across plots (square) (Correlations A in Figure 1). Open circle and square, nonsignificant; closed circle and square, significant. Negative *R* ^2^ values are shown for negative correlations

Across two spatial scales, trait correlations were generally consistent with the results across biological levels (Figure 4 and Figure S4‐S6). While the slopes of trait correlations were generally similar within and across plots, the strength of trait correlations was significantly stronger within compared to across plots (*t* tests, Table S3). However, the strength of Lchl versus SLA correlation was stronger across plots (Table S3).

### 3.3 Effects of trait variance and sample size on correlations

**FIGURE 5:**
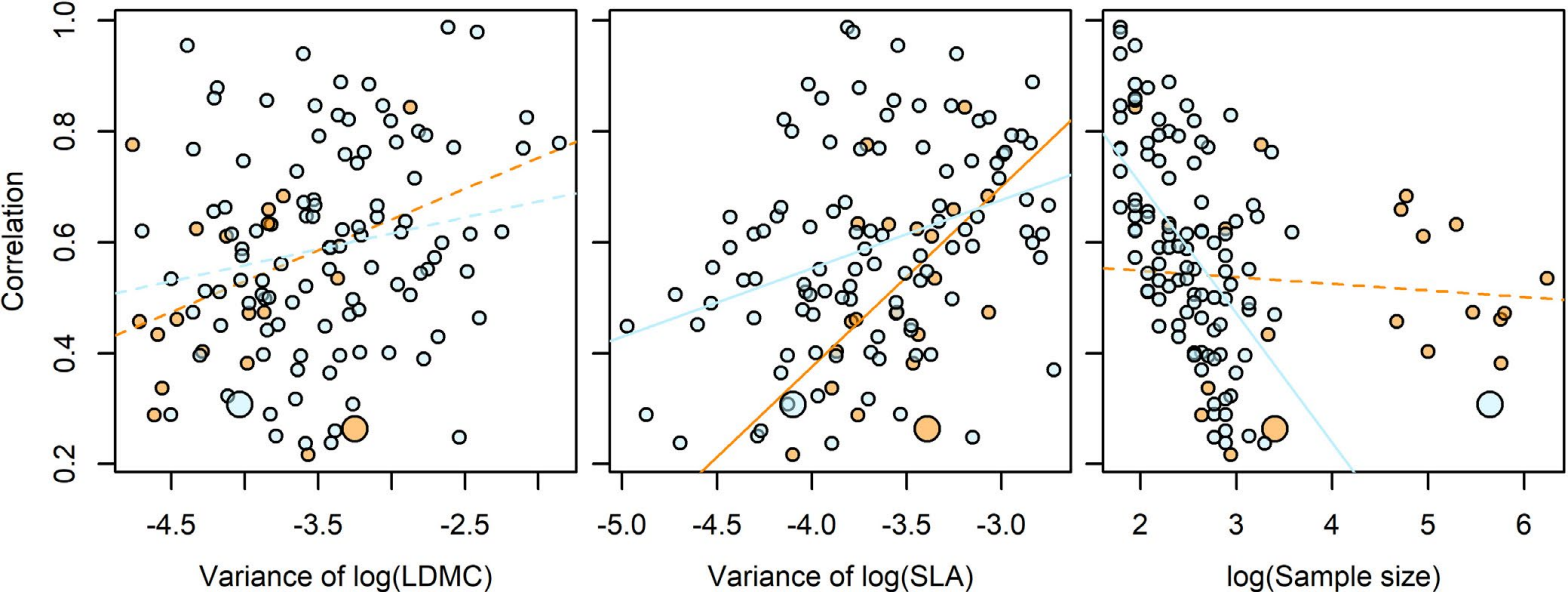
An example illustrating the effects of trait variance and sample size on the strength (*R* ^2^) of the correlation between SLA and LDMC. Small orange point, within species; small blue point, within plot; large orange point, across species; large blue point, across plots. Solid and dotted lines are significant and nonsignificant relationships by linear models

Trait correlation strength was affected by the variance of at least one trait in 10 of 26 cases, where increasing trait variance strengthened trait correlations (except for the variance of SLA on SLA versus LMR correlation, Table 1). Correlations were stronger within species than within plot in 3 cases after accounting for trait variance and sample size (Table 1). The sample size was the most important factor driving correlation strength where *R* ^2^ values of correlations were higher when fewer individuals were sampled (Table 1). The effect of sample size on correlation was stronger when analyzed within plot than species (Table 1 and Figure 5). The interactions between trait variance and levels analyzed generally were not significant except for three cases (Table 1).

**TABLE 1:** Effects of trait variance (var. Y and var. X), levels (species (sp) and plot levels), sample size (*N*), and their interactions on the strength of trait correlations by multiple regressions

Group	Trait Y	Trait X	Species number	Plot number	Var. Y	Var. X	Level (sp)	*N*	Var. Y × level (sp)	Var. X × level (sp)	*N* × level (sp)	Adjusted *R* ^2^
Leaf versus Petiole	PL	LA	17	84	−0.04	0.04	−0.12	**−0.28** ***	−0.13	0.04	**0.17** ***	.50
	PD	LT	10	30	0.01	0.03	−0.05	**−0.37** ***	−0.02	0.06	**0.28** **	.81
	SPL	SLA	11	25	0.05	**0.04** *	0.10	**−0.43** ***	0.05	−0.08	**0.32** **	.74
	PDMC	LDMC	10	25	0.03	0.04	−0.05	−0.10	−0.09	0.01	−0.05	.53
Within group	LT	LA	12	30	**0.06** ***	0.02	−0.10	**−0.36** ***	0.04	−0.10	**0.27** ***	.85
	LDMC	SLA	19	103	0.02	**0.05** **	0.02	**−0.21** ***	0.00	0.13	**0.18** ***	.39
	Lchl	SLA	18	86	**0.05** **	**0.03** *	**0.16** *	**−0.23** ***	−0.01	0.08	**0.16** ***	.50
	Lchl	LDMC	16	37	0.03	0.03	0.17	**−0.42** ***	**0.21** *	−0.05	**0.24** ***	.67
	PD	PL	12	90	**0.05** **	0.05	−0.17	**−0.24** ***	0.08	−0.14	**0.14** **	.59
	PDMC	SPL	8	20	0.06	0.01	0.09	**−0.40** ***	−0.02	0.07	**0.24** *	.81
Across groups	SLA	LA	11	24	0.03	0.00	0.00	**−0.38** ***	0.04	0.13	**0.30** **	.71
	LDMC	LA	5	36	0.02	0.02	**0.28** *	**−0.40** ***	0.13	−0.03	0.19	.73
	Lchl	LA	5	20	0.01	0.03	−0.15	**−0.50** ***	0.00	−0.03	**0.44** *	.80
	SLA	LT	17	52	0.00	0.02	**0.14** *	**−0.46** ***	0.12	−0.03	**0.34** ***	.67
	LDMC	LT	7	39	0.02	0.03	−0.02	**−0.26** ***	0.02	−0.01	**0.14** *	.75
	Lchl	LT	11	44	0.02	0.02	−0.14	**−0.44** ***	−0.04	0.05	**0.42** ***	.81
	SPL	PD	11	46	**0.05** *	0.00	−0.05	**−0.28** ***	**0.17** *	−0.06	**0.24** **	.65
	PDMC	PD	Only 2 species, thus not perform multiple regression									
	SPL	PL	9	12	0.00	0.01	0.06	−0.27	0.09	−0.12	0.09	.55
	PDMC	PL	8	18	−0.02	0.02	0.06	**−0.58** ***	−0.02	−0.13	**0.35** *	.77
Whole leaf trait	LA	LMR	7	27	0.04	**0.11** **	−0.24	**−0.43** ***	−0.11	**−0.15** *	**0.38** **	.84
	LT	LMR	Only 3 species, thus not perform multiple regression									
	SLA	LMR	8	17	**−0.05** *	**0.08** *	0.05	**−0.73** ***	0.13	−0.07	**0.60** **	.89
	LDMC	LMR	5	17	0.04	**0.12** *	−0.05	**−0.30** *	−0.13	−0.08	0.12	.75
	Lchl	LMR	5	12	0.00	0.05	−0.15	**−0.50** **	−0.10	−0.05	**0.50** *	.89
	PD	LMR	4	14	−0.01	0.13	0.19	−0.15	0.12	−0.10	−0.04	.53
	PL	LMR	10	52	**0.09** **	−0.01	−0.17	**−0.17** **	−0.13	0.01	0.04	.65
	SPL	LMR	Only 3 species, thus not perform multiple regression									
	PDMC	LMR	5	19	0.00	0.00	0.07	**−0.37** *	−0.08	0.07	0.13	0.69

Note

Bold font indicates significant effect.

* *p* < .05.

** *p* < .01.

*** *p* < .001.

## 4 DISCUSSION

In this study, we evaluated the variation and correlations of leaf lamina and petiole traits across biological and spatial scales at the seedling stage in a temperate forest. Overall, we found a large amount of trait variation (6%–63%) was explained by ITV estimated across either biological or spatial scales. Specifically, the most used leaf trait in ecological analyses, SLA, had more than 50% of its variation explained by ITV. Trait correlations varied largely across different biological and spatial levels. The strength of trait correlation was comparable between intra‐ and interspecific levels but stronger within than across plots. These results did not support the prediction that trait correlations were stronger at broad scales compared to fine scales. For the correlations at the intraspecific and within plot levels, the sample size was the most important factor driving the strength of correlations where correlations were weaker when more individuals were sampled.

### 4.1 Intraspecific variation in seedling lamina and petiole traits

A large amount of variation (around 40%, but varied among traits) in seedling leaf traits were similar to previous studies that sampled only one species across a broad spatial scale (Fajardo & Piper, 2011; Hu et al., 2015; Martin et al., 2017), or several dominant species across a broad elevational gradient (Umaña & Swenson, 2019). This result was surprising because we estimated the ITV at a local spatial extent (300 m × 300 m). The high ITV in our study likely because we sampled all individuals in the communities or a high‐trait variation at the seedling stage. Our results were consistent with another study by Messier and colleagues (Messier et al., 2010), who also estimated the ITV of all trees with dbh > 10 cm across a precipitation gradient and found 48% variation of leaf mass per area (inverse of SLA) explained by ITV. Compared to these studies, we suggest that, even in a local spatial extent, the amount of trait variation explained by ITV is nearly comparable with that at the interspecific level for plants at early life stages.

With the increasing number of studies that use trait‐based approaches to understand seedling dynamics (e.g., growth and survival) (Lebrija‐Trejos et al., 2016; Visser et al., 2016), our results suggest that we need to incorporate the ITV of leaf traits, which are likely to be the most important traits for seedling performance under limited light environments. Recent studies find or suggest that individual‐level trait values have a stronger power to predict seedling growth than species‐level ones in tropical forests (Liu et al., 2016; Umaña et al., 2018; Yang et al., 2018). The large amount of ITV in seedlings can result from multiple factors. First, seedling size is an important driver. Studies conducted in forest dynamic plots (Anderson‐Teixeira et al., 2015) usually define seedlings as individuals with height > 10 or 20 cm and dbh < 1 cm that include a wide range of sizes. The influence of plant size on leaf traits is supported by recent findings (Dayrell et al., 2018; Forrestel et al., 2015; Martin & Thomas, 2013; Mason et al., 2013; Park et al., 2019). Second, micro‐environmental heterogeneity in our plot, such as spatial variation in light availability, can also drive leaf trait variation among individuals (Rozendaal et al., 2006).

### 4.2 Trait correlations among lamina and petiole traits across biological and spatial scales

The strong correlations between lamina and petiole traits suggest that there are underlying biophysical constraints and coupled functions such as water transport and photosynthesis between the lamina and petiole (Poorter, 2009; Poorter & Rozendaal, 2008). While a larger and thicker lamina corresponded to a longer and thicker petiole, a resource acquisitive (high SLA and low LDMC) lamina was also accompanied by a resource acquisitive (high SPL and low PDMC) petiole. A similar lamina‐twig size relationship was also found by a previous study (Westoby & Wright, 2003). This functional convergence between lamina and petiole might be mechanistically linked via growth mechanisms or could be the adaptive outcome of optimal use of local environmental conditions or provided a competitive advantage than other trait combinations. For example, a low dry matter investment in petiole and lamina provided laminas with a distant position (high SPL) and larger lamina area to intercept light (high SLA), which maximized light interception and carbon assimilation rates. While lamina and petiole traits are strongly correlated, however, most studies have focused on lamina traits. For example, the leaf economics spectrum has excluded petiole traits and most studies measuring leaf traits have overlooked petiole variation (Diaz et al., 2016; Reich et al., 1997; Wright et al., 2004). Given the strong lamina–petiole trait relationships found here, it will be valuable to assess whether these relationships are consistent across species at broad scales and how these morphological traits are related to leaf physiological functions.

As we predicted, across lamina and petiole traits at different levels, leaf size, and economics traits were correlated within each trait group, but the trait correlations were weaker across trait groups. Decoupled relationships between size and economics traits have been shown in a global trait dataset and the tundra biome (Diaz et al., 2016; Thomas et al., 2020). PL was more strongly correlated with LMR than other leaf traits both within and across species, which suggests that more biomass allocated to lamina relative to petiole is driven by a shorter petiole. Although some correlations were not significant, a higher LMR was generally related to a larger, thicker, and resource conservative lamina and a shorter and resource conservative petiole. It is obvious that this whole leaf trait integrates lamina and petiole size and economics traits.

We found that the slopes of trait correlations were different across biological and spatial scales, which was consistent with previous studies (Anderegg et al., 2018). However, our results did not support recent suggestions that correlations between traits were weaker at finer scales such as within species or at a local spatial scale (Anderegg et al., 2018; Messier et al., 2017). First, we found significant and strong correlations between traits for each species or most plots (e.g., among commonly studied lamina economics traits). Second, trait correlations were stronger within than across plots. These results imply that trait combinations are also constrained within species or at a local spatial scale. Using a global dataset, Anderegg et al., (2018) also found similar correlation strength between leaf mass per area and nitrogen content per area across taxonomic scales. However, the spatial extent in our study site was far more local (<9 ha) than previous studies (Anderegg et al., 2018; Messier et al., 2010, 2017). The reason for strong trait correlations in finer scales found here might be that there was a large ITV for seedlings (e.g., trait range in one species might be larger than that across all species mean values). Compared to previous studies (Anderegg et al., 2018), trait variation in seedlings here might be mainly caused by plant growth (i.e., seedling size) rather than environmental gradients and the changes of functional traits might converge more along with ontogenetic stages than environmental gradients.

### 4.3 Correlations, trait variance, and sample size within species and plots

While there was substantial variation in the strength of trait correlations among species and plots, we used multiple regressions to evaluate the underlying factors. The increased correlation strength with increased trait variance was consistent with our prediction and suggestion in previous studies (Anderegg et al., 2018; Messier et al., 2017). For example, the variation of leaf lifespan among species determined the degree of trait correlations between leaf economics spectrum traits (Wright et al., 2005). However, relative to trait variance, sample size was the most important factor where trait correlations decreased when more individuals were sampled (Anderegg et al., 2018; Wright et al., 2005). These previous studies rarely evaluated the effect of sample size. Wright and colleagues found that the slopes from trait correlations within one site were similar to those across global scales when more species were sampled (Wright et al., 2005). The potential reason might be that the environmental heterogeneity increased when more individuals were sampled, and traits might have specific responses to environmental gradients (Umaña & Swenson, 2019). We also found that the effects of sample size on trait correlations were different between species and plot levels (Figure 5). The reason underlying this difference might be that the driver of trait variation within a species was different from that within a plot: Environment and seedling size influenced intraspecific trait variation while species identity and seedling size were the causes of trait variation within a plot. This environment‐driven trait variation within a species might increase constraints of trait combinations, thus a stronger correlation than that within a plot (Table 1) (Delhaye et al., 2020; Dwyer & Laughlin, 2017). Finally, our results indicated that trait variance and sample size together explained a large amount of variation (39%–89%, Table 1) in the strength of trait correlations.

## 5 CONCLUSION

Using a large dataset of 10 lamina and petiole traits for seedlings in a temperate forest, we found about 40% trait variation could be explained within species. A large amount of ITV suggests the need to consider individual‐level traits when we explore ecological questions using trait‐based approaches in seedlings. The tightly correlated lamina and petiole traits implied the convergent functions between both leaf parts. Disagreeing with recent studies, we found that trait correlations were not stronger in broader compared to finer scales (especially across vs. within plots) (Anderegg et al., 2018; Messier et al., 2017). Finally, we found the most amount of variation in the strength of trait correlations within species and plots could be explained by trait variance and sample size. In conclusion, individual‐level traits are important for us to understand plant demography and community assembly at the seedling stage.

## CONFLICT OF INTEREST

The authors declare no conflicts of interest.

## AUTHOR CONTRIBUTIONS

**Feng Jiang:** Conceptualization (lead); Formal analysis (lead); Investigation (lead); Methodology (lead); Visualization (lead); Writing‐original draft (lead); Writing‐review & editing (lead). **Marc W Cadotte:** Formal analysis (supporting); Investigation (supporting); Resources (supporting); Supervision (lead); Visualization (supporting); Writing‐original draft (supporting); Writing‐review & editing (lead). **Guangze Jin:** Conceptualization (supporting); Data curation (lead); Formal analysis (supporting); Funding acquisition (lead); Investigation (supporting); Resources (lead); Supervision (lead); Writing‐original draft (supporting); Writing‐review & editing (supporting).

## Supporting information

Supplementary Material

## Data Availability

Dataset is uploaded in the website DRYAD https://doi.org/10.5061/dryad.18931zcwq.
