# Supplementary material for: Individual‐level leaf trait variation and correlation across biological and spatial scales
Source: Ecol Evol. 2021 Mar 18;11(10):5344–54. doi: 10.1002/ece3.7425 (PMC8131770; doi:10.1002/ece3.7425)

**Table S1.** The biological information of species sampled in our study.

| Family | Genera | Species code | Individual number |
| --- | --- | --- | --- |
| Adoxaceae | *Sambucus* | SamWil | 1 |
|  | *Viburnum* | VibBur | 14 |
|  |  | VibSar | 7 |
| Araliaceae | *Aralia* | AraEla | 3 |
|  | *Eleutherococcus* | EleSen | 328 |
| Berberidaceae | *Berberis* | BerPoi | 19 |
| Betulaceae | *Corylus* | CorMan | 148 |
| Caprifoliaceae | *Lonicera* | LonGen | 337 |
| Celastraceae | *Euonymus* | EuoVer | 318 |
| Fagaceae | *Quercus* | QueMon | 2 |
| Hydrangeaceae | *Deutzia* | DeuGla | 520 |
|  | *Philadelphus* | PhiSch | 243 |
| Leguminosae | *Albizia* | MaaAmu | 10 |
| Malvaceae | *Tilia* | TilAmu | 17 |
|  |  | TilMan | 5 |
| Oleaceae | *Fraxinus* | FraMan | 200 |
|  | *Syringa* | SyrRet | 119 |
| Rhamnaceae | *Rhamnus* | RhaDav | 2 |
| Rosaceae | *Prunus* | PruPad | 5 |
|  | *Rosa* | RosAci | 5 |
|  | *Sorbaria* | SorSor | 31 |
|  | *Spiraea* | SpiSal | 36 |
| Salicaceae | *Populus* | PopDav | 1 |
| Sapindaceae | *Acer* | AceMon | 110 |
|  |  | AceTeg | 144 |
|  |  | AceUku | 15 |
| Saxifragaceae | *Ribes* | RibBur | 19 |
|  |  | RibMan | 114 |
| Ulmaceae | *Ulmus* | UlmDav | 7 |
|  |  | UlmLac | 26 |

**Table S2.** SMA regression results for each and across species. N.species P/N/NO indicates that species number of positively significant/negatively significant/non-significant trait Y ~ trait X correlations, the common slope is also tested (no common slopes for all trait correlations). We also performed SMA regression across species using the species mean values. We used the t-test to compare the differences of slope and *R*^2^ between intraspecific- and species-levels, with no weight (Null), with the variance of trait Y as a weight (Var1), with the variance of trait X (Var2) and sample size (N). Significant (p<0.05) result is indicated as a bold font.

|  |  |  | Slope | | R2 | | N.species  P/N/NO | Common slope | Species level | | Slope test (p values) | | | | *R*^2^ test (p values) | | | |
| --- | --- | --- | --- | --- | --- | --- | --- | --- | --- | --- | --- | --- | --- | --- | --- | --- | --- | --- |
| Group | Trait Y | Trait X | Range | Mean ± SD | Range | Mean ± SD |  |  | Slope | *R*^2^ | Null | Var1 | Var2 | N | Null | Var1 | Var2 | N |
| Leaf vs. Petiole | PL | LA | -1.38~0.97 | 0.56±0.48 | 0~0.78 | 0.34±0.23 | 17/0/4 | **0.64** | -1.65 | 0.00 | 0.000 | 0.000 | 0.000 | 0.000 | 0.000 | 0.000 | 0.000 | 0.000 |
|  | PD | LT | -3.66~3.97 | 1.79±1.8 | 0~0.57 | 0.09±0.13 | 10/0/12 | **2.12** | 1.85 | 0.06 | 0.884 | 0.741 | 0.634 | 0.751 | 0.241 | 0.069 | 0.034 | 0.443 |
|  | SPL | SLA | 0.62~2.9 | 1.8±0.63 | 0.08~0.76 | 0.34±0.2 | 11/0/5 | **1.80** | **2.66** | **0.47** | 0.000 | 0.003 | 0.000 | 0.000 | 0.022 | 0.533 | 0.018 | 0.000 |
|  | PDMC | LDMC | 0.68~2.5 | 1.3±0.46 | 0.03~0.79 | 0.34±0.19 | 10/0/6 | **1.26** | **1.31** | **0.36** | 0.922 | 0.009 | 0.437 | 0.704 | 0.618 | 0.095 | 0.298 | 0.033 |
| Within group | LT | LA | -0.68~0.51 | 0.25±0.24 | 0~0.49 | 0.13±0.11 | 12/0/10 | **0.31** | **0.22** | **0.22** | 0.617 | 0.925 | 0.390 | 0.011 | 0.001 | 0.362 | 0.060 | 0.000 |
|  | LDMC | SLA | -1.48~-0.54 | -0.81±0.24 | 0.05~0.84 | 0.46±0.21 | 0/19/3 | **-0.79** | **-1.07** | **0.26** | 0.000 | 0.040 | 0.000 | 0.000 | 0.000 | 0.001 | 0.000 | 0.000 |
|  | Lchl | SLA | 0.42~1.23 | 0.88±0.2 | 0.1~0.75 | 0.45±0.17 | 18/0/4 | **0.92** | **1.08** | **0.71** | 0.000 | 0.001 | 0.000 | 0.000 | 0.000 | 0.000 | 0.000 | 0.000 |
|  | Lchl | LDMC | -1.56~0.83 | -1.07±0.51 | 0~0.85 | 0.26±0.21 | 0/16/6 | **-1.19** | **-1.01** | **0.18** | 0.610 | 0.192 | 0.330 | 0.003 | 0.088 | 0.078 | 0.333 | 0.441 |
|  | PD | PL | -1.43~3.67 | 0.88±1.02 | 0~0.57 | 0.17±0.17 | 12/0/9 | **1.06** | 0.25 | 0.11 | 0.010 | 0.002 | 0.004 | 0.000 | 0.138 | 0.044 | 0.066 | 0.777 |
|  | PDMC | SPL | -1.76~0.32 | -0.57±0.5 | 0~0.61 | 0.16±0.19 | 0/8/8 | **-0.54** | 0.53 | 0.02 | 0.000 | 0.000 | 0.000 | 0.000 | 0.010 | 0.001 | 0.009 | 0.086 |
| Across group | SLA | LA | -0.53~0.38 | -0.31±0.22 | 0~0.59 | 0.13±0.15 | 0/11/11 | **-0.37** | -0.25 | 0.09 | 0.219 | 0.059 | 0.358 | 0.000 | 0.201 | 0.086 | 0.082 | 0.746 |
|  | LDMC | LA | -0.37~0.61 | 0.12±0.3 | 0~0.82 | 0.08±0.2 | 3/2/17 | **0.29** | 0.27 | 0.02 | 0.038 | 0.515 | 0.004 | 0.051 | 0.173 | 0.053 | 0.053 | 0.589 |
|  | Lchl | LA | -0.5~0.42 | -0.1±0.32 | 0~0.22 | 0.04±0.05 | 1/4/17 | **-0.34** | -0.27 | 0.05 | 0.026 | 0.030 | 0.014 | 0.010 | 0.102 | 0.029 | 0.790 | 0.000 |
|  | SLA | LT | -2.37~-0.6 | -1.2±0.4 | 0.01~0.84 | 0.27±0.21 | 0/17/5 | **-1.17** | **-1.12** | **0.20** | 0.351 | 0.223 | 0.067 | 0.186 | 0.124 | 0.043 | 0.017 | 0.233 |
|  | LDMC | LT | -0.89~2.79 | 0.54±0.94 | 0~0.42 | 0.07±0.11 | 6/1/15 | **0.92** | -1.20 | 0.09 | 0.000 | 0.000 | 0.000 | 0.000 | 0.543 | 0.677 | 0.901 | 0.000 |
|  | Lchl | LT | -1.54~0.99 | -0.68±0.83 | 0~0.42 | 0.12±0.13 | 0/11/11 | **-1.08** | -1.21 | 0.11 | 0.006 | 0.022 | 0.000 | 0.063 | 0.601 | 0.615 | 0.054 | 0.814 |
|  | SPL | PD | -1.41~-0.76 | -1.04±0.17 | 0.01~0.78 | 0.32±0.21 | 0/11/5 | **-1.06** | **-1.61** | **0.19** | 0.000 | 0.000 | 0.000 | 0.000 | 0.022 | 0.001 | 0.005 | 0.002 |
|  | PDMC | PD | -1.93~0.48 | -0.45±0.66 | 0~0.52 | 0.08±0.14 | 0/1/15 | **-0.54** | **-0.85** | **0.33** | 0.028 | 0.468 | 0.002 | 0.002 | 0.000 | 0.000 | 0.000 | 0.000 |
|  | SPL | PL | -2.24~2.27 | -0.33±1.3 | 0.02~0.75 | 0.24±0.25 | 1/8/7 | **-1.14** | **0.42** | **0.28** | 0.036 | 0.000 | 0.001 | 0.000 | 0.531 | 0.272 | 0.551 | 0.009 |
|  | PDMC | PL | -2.05~0.6 | -0.49±0.7 | 0~0.67 | 0.16±0.2 | 2/6/8 | **-0.62** | -0.22 | 0.04 | 0.139 | 0.002 | 0.527 | 0.227 | 0.030 | 0.002 | 0.080 | 0.185 |
| Whole leaf trait | LA | LMR | -11.08~32.54 | 10.09±9.67 | 0~0.6 | 0.16±0.17 | 7/0/9 | **9.97** | **12.41** | **0.17** | 0.352 | 0.704 | 0.003 | 0.179 | 0.848 | 0.023 | 0.006 | 0.026 |
|  | LT | LMR | -16.46~11.23 | 2.11±6.16 | 0~0.32 | 0.09±0.08 | 3/0/13 | **3.34** | **2.76** | **0.26** | 0.679 | 0.109 | 0.356 | 0.416 | 0.000 | 0.000 | 0.000 | 0.000 |
|  | SLA | LMR | -14.64~5.53 | -4.69±4.75 | 0~0.45 | 0.13±0.16 | 0/8/8 | **-4.00** | **-3.08** | **0.24** | 0.198 | 0.027 | 0.947 | 0.101 | 0.015 | 0.243 | 0.005 | 0.000 |
|  | LDMC | LMR | -5.99~10.23 | 2.46±4.13 | 0~0.33 | 0.12±0.11 | 5/0/11 | **3.04** | 3.31 | 0.05 | 0.421 | 0.923 | 0.034 | 0.422 | 0.024 | 0.013 | 0.139 | 0.117 |
|  | Lchl | LMR | -12.87~7.28 | -2.39±5.23 | 0~0.25 | 0.08±0.07 | 2/3/11 | **-4.03** | **-3.35** | **0.19** | 0.472 | 0.578 | 0.053 | 0.116 | 0.000 | 0.000 | 0.000 | 0.000 |
|  | PD | LMR | -16.38~22.75 | 2.39±10.85 | 0~0.57 | 0.12±0.18 | 4/0/12 | **7.05** | -5.09 | 0.01 | 0.015 | 0.025 | 0.003 | 0.024 | 0.031 | 0.051 | 0.131 | 0.272 |
|  | PL | LMR | -17.67~-2.77 | -8.58±4.35 | 0~0.86 | 0.25±0.22 | 0/10/6 | **-6.57** | **-19.58** | **0.68** | 0.000 | 0.000 | 0.000 | 0.000 | 0.000 | 0.000 | 0.000 | 0.000 |
|  | SPL | LMR | -30.27~16.06 | -2.96±11.49 | 0~0.32 | 0.06±0.11 | 1/2/13 | **-7.23** | **-8.21** | **0.28** | 0.088 | 0.754 | 0.008 | 0.007 | 0.000 | 0.000 | 0.000 | 0.000 |
|  | PDMC | LMR | -9.41~13.18 | 0.88±6.03 | 0~0.47 | 0.1±0.14 | 4/1/11 | **3.83** | -4.33 | 0.00 | 0.004 | 0.400 | 0.001 | 0.000 | 0.020 | 0.010 | 0.017 | 0.021 |

**Table S3.** SMA regression results for each and across plots. N. plot P/N/NO indicates that plot number of positively significant/negatively significant/non-significant trait Y ~ trait X correlations, common slope is also tested (no common slopes for all trait correlations). We also performed SMA regression across plots using the plot mean values. We used the t-test to compare the differences of slope and *R*^2^ between within plot- and plot-levels, with no weight (Null), with the variance of trait Y as a weight (Var1), with the variance of trait X (Var2) and sample size (N). Significant (p<0.05) result is indicated as a bold font.

|  |  |  | Slope | | R2 | | N.plot  P/N/NO | Common  slope | Species level | | Slope test (p values) | | | | *R*^2^ test (p values) | | | |
| --- | --- | --- | --- | --- | --- | --- | --- | --- | --- | --- | --- | --- | --- | --- | --- | --- | --- | --- |
| Group | Trait Y | Trait X | Range | Mean ± SD | Range | Mean ± SD |  |  | Slope | *R*^2^ | Null | Var1 | Var2 | N | Null | Var1 | Var2 | N |
| Leaf vs. Petiole | PL | LA | -3.49~5.76 | 1.1±1.52 | 0~0.94 | 0.34±0.28 | 81/3/126 | **1.49** | **1.69** | **0.13** | 0.000 | 0.000 | 0.000 | 0.000 | 0.000 | 0.000 | 0.000 | 0.000 |
|  | PD | LT | -9~8.28 | 0.86±3.1 | 0~0.85 | 0.17±0.18 | 29/1/184 | **2.38** | 2.00 | 0.00 | 0.000 | 0.000 | 0.000 | 0.000 | 0.000 | 0.000 | 0.000 | 0.000 |
|  | SPL | SLA | -3.34~9.17 | 2.59±2 | 0~0.94 | 0.35±0.29 | 25/0/60 | **2.54** | **2.76** | **0.18** | 0.440 | 0.080 | 0.000 | 0.133 | 0.000 | 0.000 | 0.000 | 0.000 |
|  | PDMC | LDMC | -4.33~4 | 0.84±1.48 | 0~0.95 | 0.34±0.29 | 25/0/56 | **1.33** | **1.84** | **0.23** | 0.000 | 0.000 | 0.000 | 0.000 | 0.001 | 0.000 | 0.000 | 0.001 |
| Within group | LT | LA | -0.91~0.59 | 0.07±0.26 | 0~0.91 | 0.17±0.2 | 26/4/183 | **0.22** | **0.29** | **0.03** | 0.000 | 0.000 | 0.000 | 0.000 | 0.000 | 0.000 | 0.000 | 0.000 |
|  | LDMC | SLA | -2.79~2.14 | -1.05±0.75 | 0~0.99 | 0.38±0.26 | 1/102/106 | **-1.14** | **-1.03** | **0.31** | 0.745 | 0.000 | 0.785 | 0.189 | 0.000 | 0.000 | 0.000 | 0.000 |
|  | Lchl | SLA | -2.02~1.89 | 0.73±0.69 | 0~0.96 | 0.32±0.25 | 85/1/128 | **0.90** | **0.99** | **0.56** | 0.000 | 0.052 | 0.000 | 0.000 | 0.000 | 0.000 | 0.000 | 0.000 |
|  | Lchl | LDMC | -12.79~1.92 | -0.33±1.22 | 0~0.88 | 0.18±0.2 | 11/26/172 | **-0.78** | **-0.96** | **0.08** | 0.000 | 0.005 | 0.000 | 0.000 | 0.000 | 0.000 | 0.000 | 0.000 |
|  | PD | PL | -1.67~1.63 | 0.32±0.31 | 0~0.95 | 0.36±0.27 | 90/0/121 | **0.35** | **0.34** | **0.36** | 0.302 | 0.011 | 0.000 | 0.072 | 0.623 | 0.000 | 0.001 | 0.562 |
|  | PDMC | SPL | -1.08~2.48 | 0.4±0.64 | 0~0.85 | 0.28±0.26 | 17/3/64 | **0.61** | **0.69** | **0.11** | 0.000 | 0.626 | 0.000 | 0.000 | 0.000 | 0.000 | 0.000 | 0.000 |
| Across group | SLA | LA | -1.4~0.62 | -0.1±0.28 | 0~0.87 | 0.17±0.19 | 2/22/189 | **-0.23** | -0.28 | 0.01 | 0.000 | 0.000 | 0.000 | 0.000 | 0.000 | 0.000 | 0.000 | 0.000 |
|  | LDMC | LA | -0.85~0.82 | 0.07±0.32 | 0~0.99 | 0.2±0.21 | 27/9/172 | **0.27** | **0.29** | **0.02** | 0.000 | 0.000 | 0.000 | 0.000 | 0.000 | 0.000 | 0.000 | 0.000 |
|  | Lchl | LA | -0.54~1.02 | 0.04±0.26 | 0~0.87 | 0.15±0.18 | 12/8/193 | **0.20** | 0.28 | 0.00 | 0.000 | 0.000 | 0.000 | 0.000 | 0.000 | 0.000 | 0.000 | 0.000 |
|  | SLA | LT | -3.57~3.42 | -0.6±1.16 | 0~0.91 | 0.22±0.23 | 4/48/162 | **-1.05** | **-0.97** | **0.18** | 0.000 | 0.006 | 0.000 | 0.000 | 0.011 | 0.000 | 0.000 | 0.327 |
|  | LDMC | LT | -4.47~4.34 | -0.82±1.28 | 0~0.88 | 0.21±0.21 | 2/37/170 | **-1.23** | **-1.00** | **0.02** | 0.045 | 0.691 | 0.000 | 0.049 | 0.000 | 0.000 | 0.000 | 0.000 |
|  | Lchl | LT | -3.52~4.13 | -0.64±1 | 0~0.95 | 0.21±0.22 | 1/43/170 | **-0.95** | **-0.96** | **0.13** | 0.000 | 0.000 | 0.000 | 0.000 | 0.000 | 0.000 | 0.000 | 0.000 |
|  | SPL | PD | -2.24~3.12 | -0.96±0.75 | 0~0.97 | 0.52±0.29 | 0/46/39 | **-1.12** | **-1.35** | **0.24** | 0.000 | 0.004 | 0.000 | 0.000 | 0.000 | 0.000 | 0.000 | 0.000 |
|  | PDMC | PD | -1.63~1.07 | -0.48±0.6 | 0~0.86 | 0.32±0.27 | 0/24/60 | **-0.64** | **-0.93** | **0.10** | 0.000 | 0.000 | 0.000 | 0.000 | 0.000 | 0.000 | 0.000 | 0.000 |
|  | SPL | PL | -3.02~2.06 | -0.5±0.94 | 0~0.78 | 0.25±0.22 | 4/8/73 | **-0.73** | **-0.51** | **0.03** | 0.905 | 0.327 | 0.000 | 0.385 | 0.000 | 0.000 | 0.000 | 0.000 |
|  | PDMC | PL | -1.23~1.85 | -0.18±0.56 | 0~0.95 | 0.28±0.26 | 2/16/66 | **-0.42** | **-0.35** | **0.02** | 0.006 | 0.016 | 0.000 | 0.008 | 0.000 | 0.000 | 0.000 | 0.000 |
| Whole leaf trait | LA | LMR | -42.76~54.58 | 8.29±15.11 | 0~0.97 | 0.34±0.26 | 26/1/58 | **11.82** | **7.44** | **0.07** | 0.608 | 0.402 | 0.275 | 0.205 | 0.000 | 0.000 | 0.000 | 0.000 |
|  | LT | LMR | -11.33~12.11 | 1.1±3.49 | 0~0.88 | 0.19±0.23 | 9/3/73 | **2.53** | **2.27** | **0.05** | 0.003 | 0.073 | 0.000 | 0.001 | 0.000 | 0.000 | 0.000 | 0.000 |
|  | SLA | LMR | -8.55~6.91 | -1.54±2.99 | 0~0.96 | 0.29±0.27 | 3/14/68 | **-2.57** | **-2.13** | **0.04** | 0.069 | 0.937 | 0.000 | 0.065 | 0.000 | 0.000 | 0.000 | 0.000 |
|  | LDMC | LMR | -5.41~16.09 | 1.63±3.58 | 0~0.86 | 0.25±0.25 | 16/1/65 | **3.00** | **2.20** | **0.03** | 0.150 | 0.532 | 0.001 | 0.269 | 0.000 | 0.000 | 0.000 | 0.000 |
|  | Lchl | LMR | -7.67~11.39 | -0.45±3.31 | 0~0.75 | 0.2±0.21 | 3/9/73 | **-2.50** | **-2.07** | **0.02** | 0.000 | 0.000 | 0.000 | 0.000 | 0.000 | 0.000 | 0.000 | 0.000 |
|  | PD | LMR | -30.99~32.23 | 0.25±9.42 | 0~0.85 | 0.25±0.24 | 3/11/71 | **-6.07** | **-4.36** | **0.06** | 0.000 | 0.000 | 0.000 | 0.000 | 0.000 | 0.000 | 0.000 | 0.000 |
|  | PL | LMR | -54.37~14.73 | -9.79±8.02 | 0~0.98 | 0.55±0.3 | 0/52/33 | **-10.71** | **-11.52** | **0.30** | 0.049 | 0.472 | 0.000 | 0.027 | 0.000 | 0.000 | 0.000 | 0.000 |
|  | SPL | LMR | -30.55~21.25 | -1.1±9.52 | 0~0.7 | 0.2±0.19 | 4/3/78 | **-6.72** | 5.89 | 0.01 | 0.000 | 0.000 | 0.000 | 0.000 | 0.000 | 0.000 | 0.000 | 0.000 |
|  | PDMC | LMR | -10.39~29.58 | 1.81±5.56 | 0~0.89 | 0.26±0.25 | 16/3/65 | **3.99** | **4.05** | **0.13** | 0.000 | 0.168 | 0.000 | 0.001 | 0.000 | 0.000 | 0.000 | 0.000 |

**Fig. S1.** Topography of the 9-ha forest dynamics plot (left panel) and distributions of 283 4 m^2^ sampled seedlings plots (right panel, red squares).


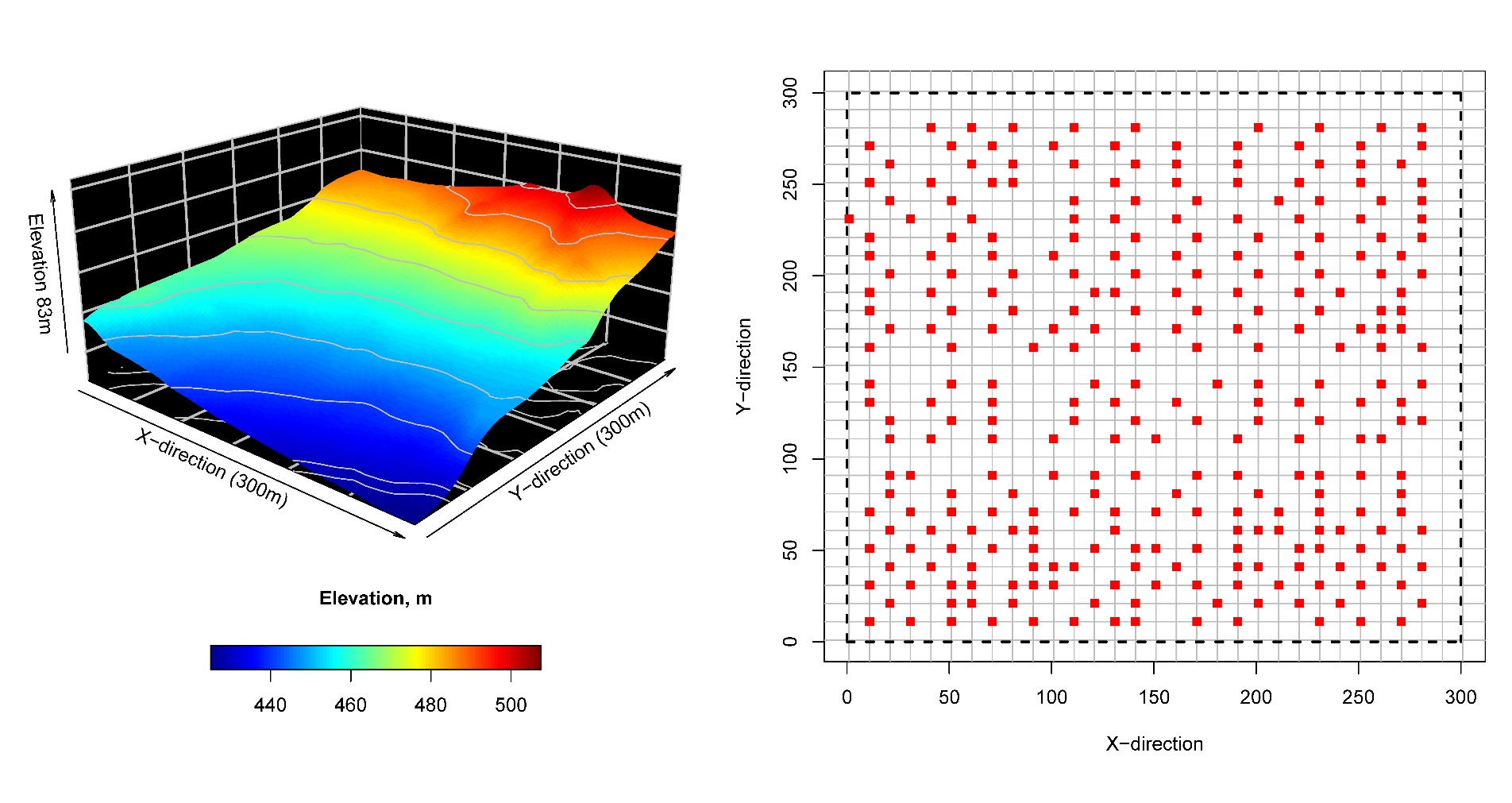


**Fig. S2.** Correlations between lamina traits across trait groups within species (blue lines) and across species (purple lines) (*Correlations C in Fig. 1*). Grey circle, values of seedling individuals for all species; purple circle, mean values of species; dashed line, non-significant; solid line, significant. Correlations are generated using standardized major axis (SMA) regressions.


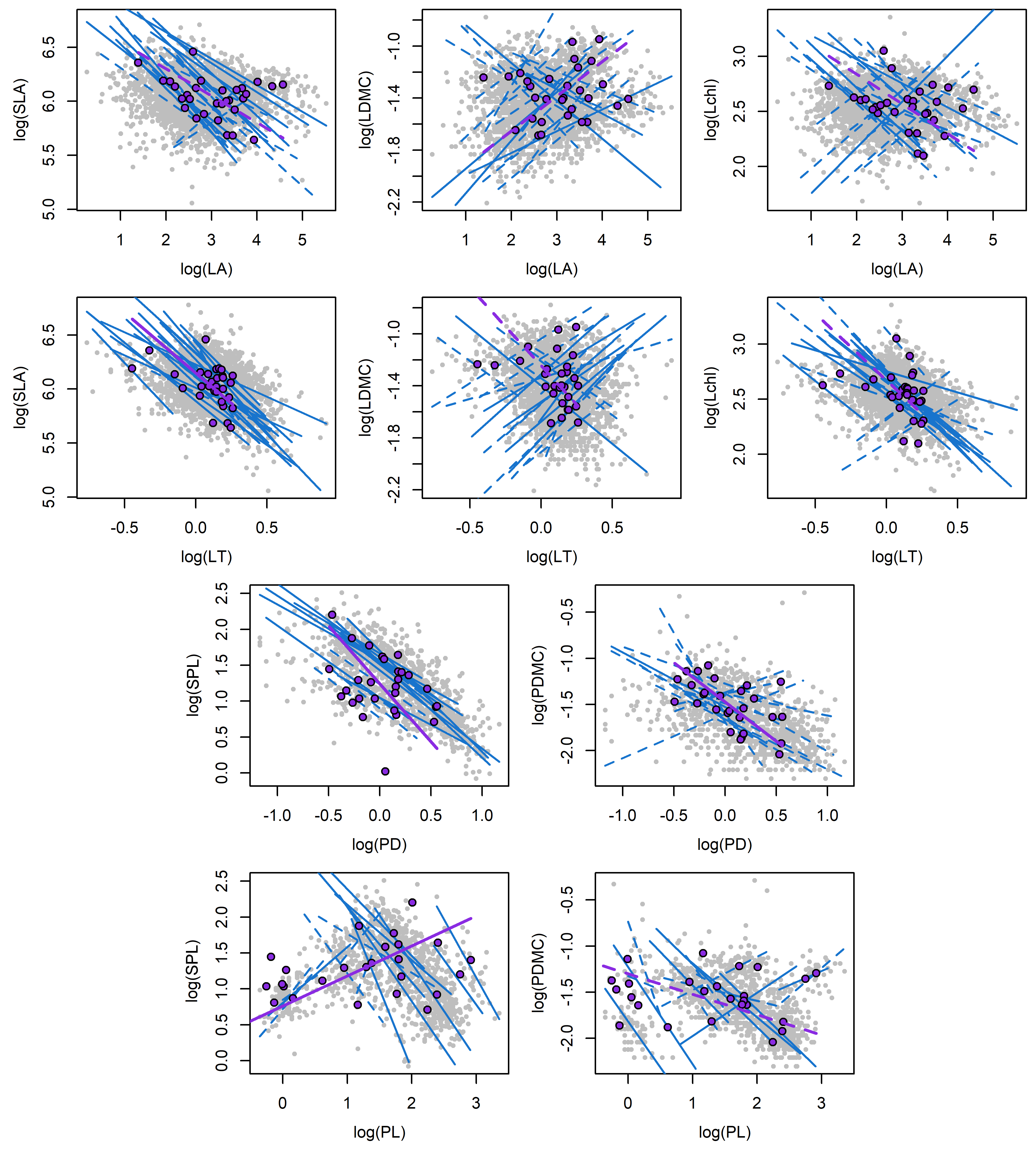


**Fig. S3.** Correlations between LMR and other traits within species (black lines) and across species (purple lines) (*Correlations D in Fig. 1*). Grey circle, values of seedling individuals for all species; purple circle, mean values of species; dashed line, non-significant; solid line, significant. Correlations are generated using standardized major axis (SMA) regressions.


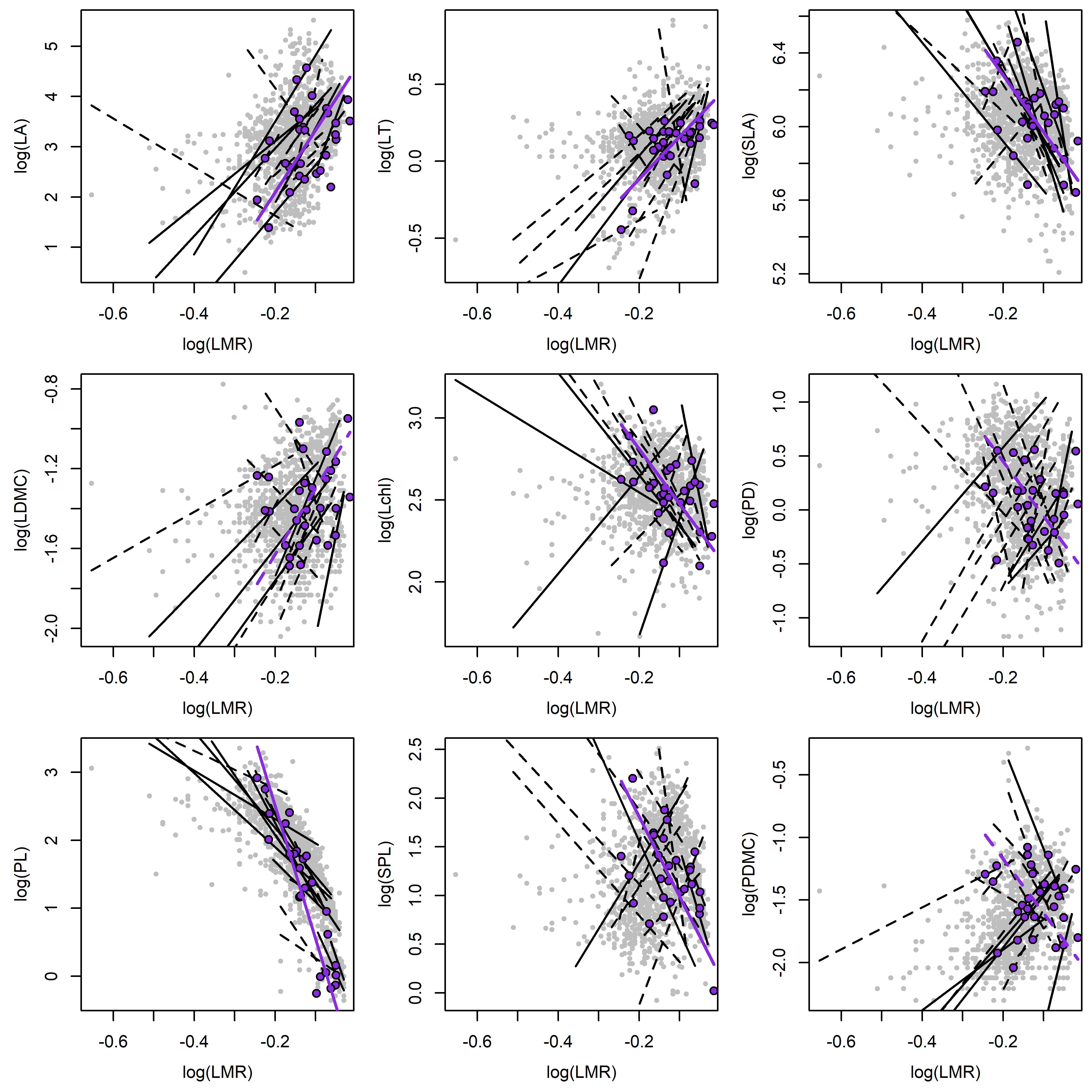


**Fig. S4.** Slopes and *R*^2^ values of standardized major axis (SMA) regressions between traits within trait groups within plots (circle) and across plots (square) (*Correlations B in Fig. 1*). Open circle and square, non-significant; closed circle and square, significant. Negative *R*^2^ values are shown for negative correlations.


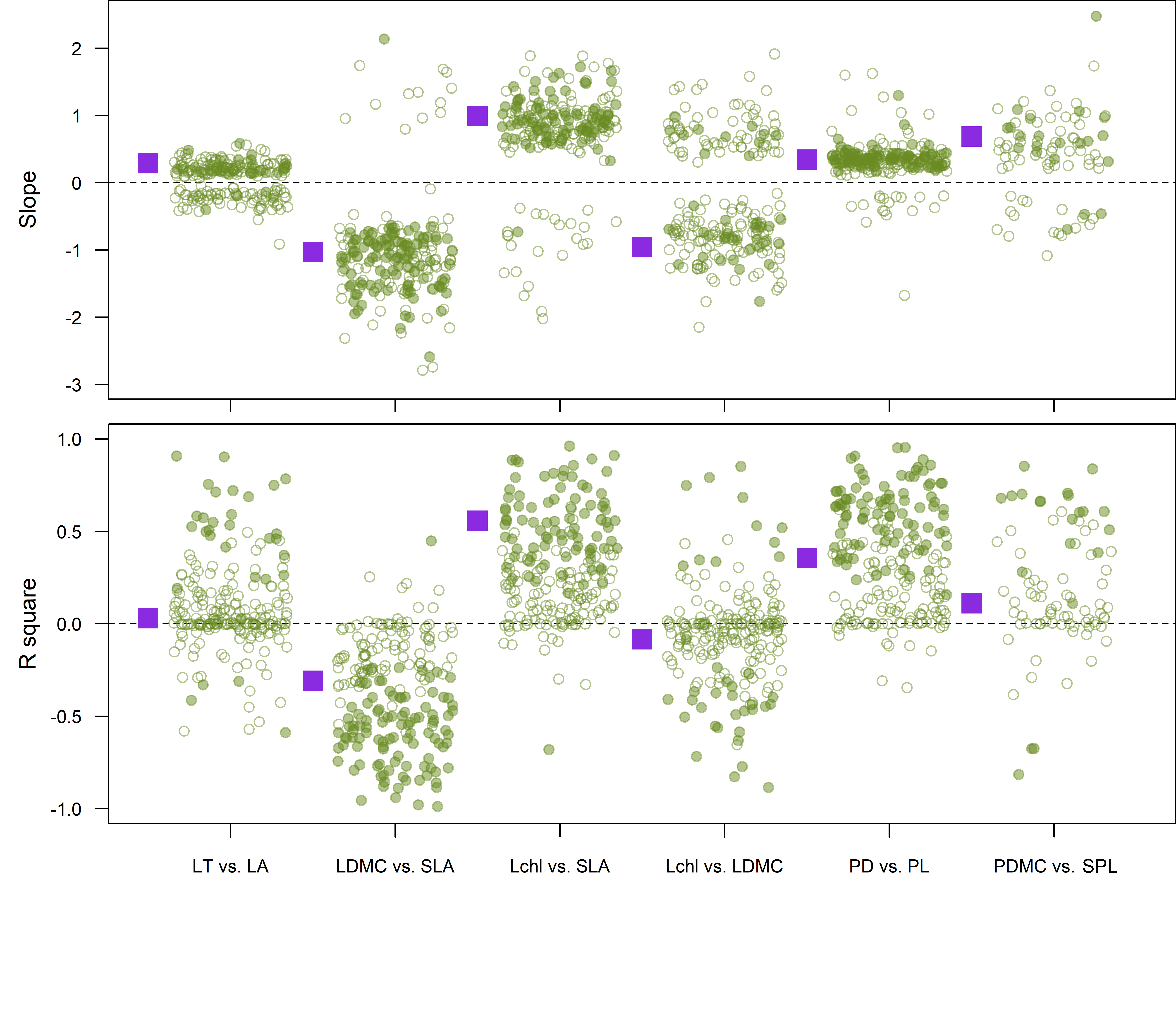


**Fig. S5.** Slopes and *R*^2^ values of standardized major axis (SMA) regressions between lamina traits across trait groups within plots (circle) and across plots (square) (*Correlations C in Fig. 1*). Open circle and square, non-significant; closed circle and square, significant. Negative *R*^2^ values are shown for negative correlations.


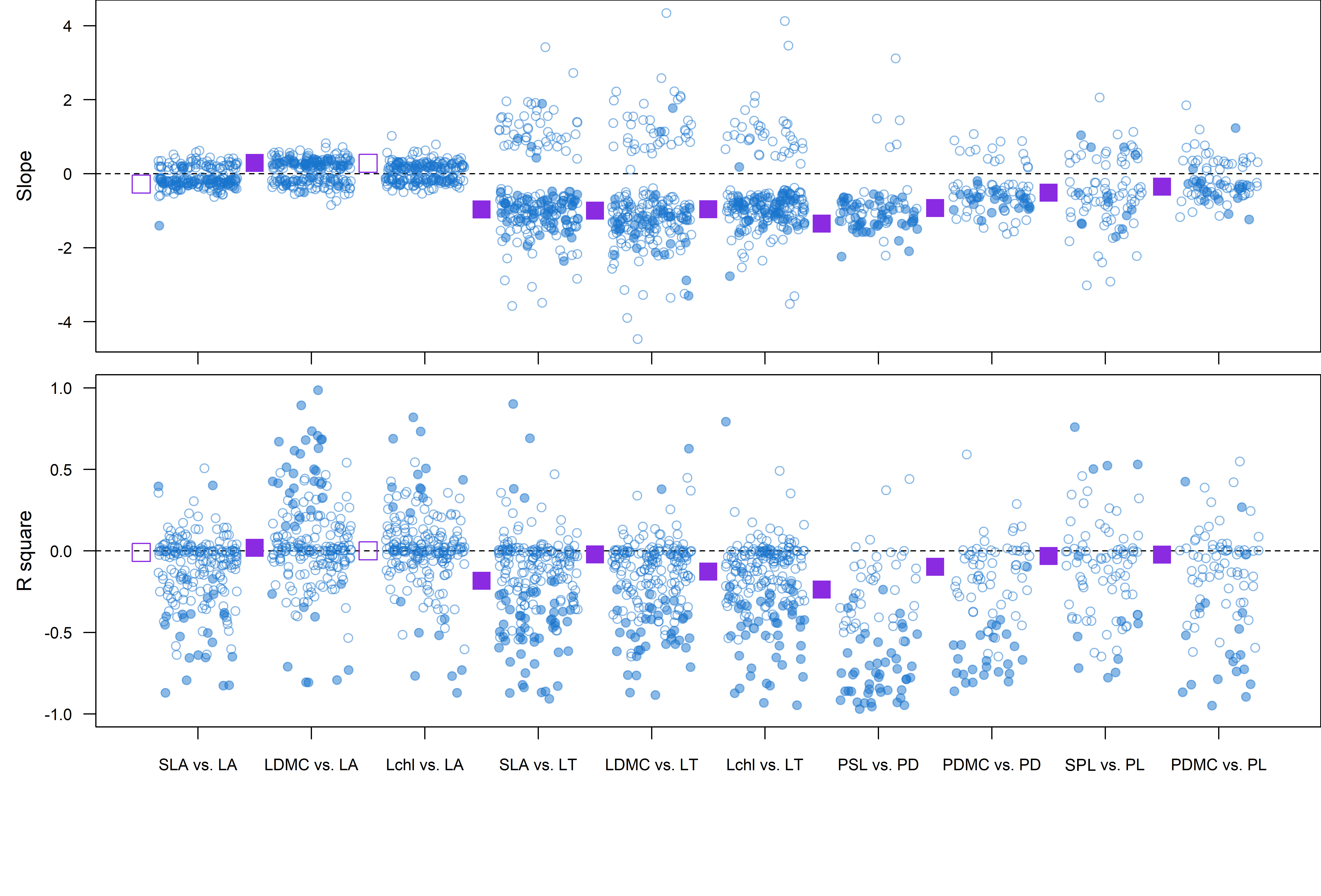


**Fig. S6.** Slopes and *R*^2^ values of standardized major axis (SMA) regressions between LMR and other traits within plots (circle) and across plots (square) (*Correlations D in Fig. 1*). Open circle and square, non-significant; closed circle and square, significant. Negative *R*^2^ values are shown for negative correlations.


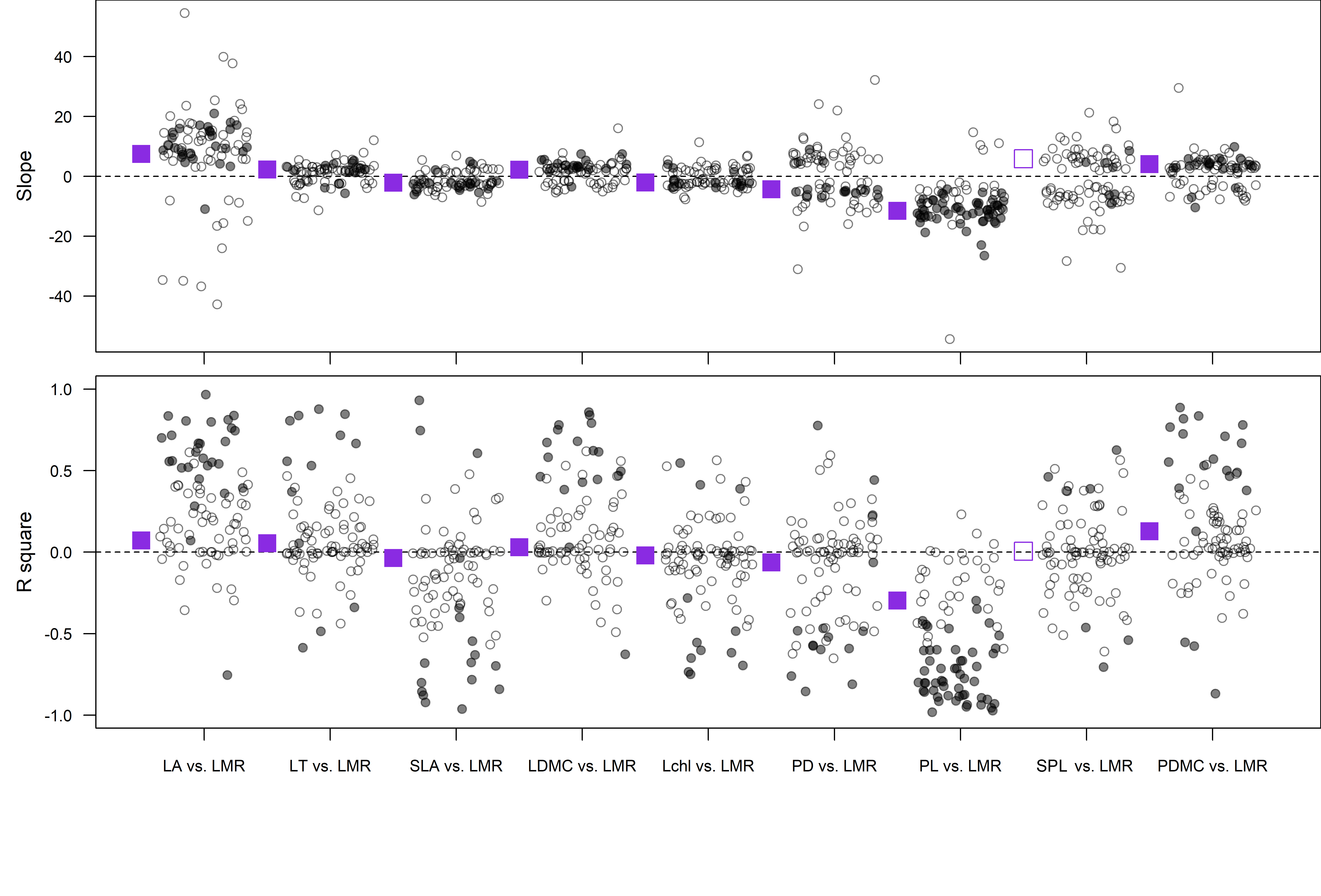

Supplement: Supplementary file 1 — Supplementary Material [file ECE3-11-5344-s001.docx]
